# The pro‐inflammatory effect of NR4A3 in osteoarthritis

**DOI:** 10.1111/jcmm.14804

**Published:** 2019-11-07

**Authors:** Chiyuan Ma, Lingyun Wu, Lu Song, Yuzhe He, Safwat Adel Abdo Moqbel, Shigui Yan, Kunkun Sheng, Haobo Wu, Jisheng Ran, Lidong Wu

**Affiliations:** ^1^ Department of Orthopedics Surgery The 2nd Affiliated Hospital Zhejiang University School of Medicine Hangzhou China; ^2^ Department of Radiation Oncology The 1st Affiliated Hospital Zhejiang University School of Medicine Hangzhou China; ^3^ Department of Stomatology Huaihe Hospital of Henan University Kaifeng China

**Keywords:** chondrocyte, NF‐κB, NR4A3, osteoarthritis

## Abstract

NR4A3 is a member of nuclear receptor subfamily 4, which is an important regulator of cellular function and inflammation. In this study, high expression of NR4A3 in human osteoarthritis (OA) cartilage was firstly observed. To explore the relationship between NR4A3 and OA, we used a lentivirus overexpression system to simulate its high expression and study its role in OA. Additionally, siRNA‐mediated knockdown of NR4A3 was used to confirm the findings of overexpression experiments. The results showed the stimulatory effect of IL‐1β on cartilage matrix‐degrading enzyme expression such as MMP‐3, 9, INOS and COX‐2 was enhanced in NR4A3‐overexpressed chondrocytes and decreased in NR4A3‐knockdown chondrocytes at both mRNA and protein levels, while IL‐1β‐induced chondrocyte‐specific gene (collagen 2 and SOX‐9) degradation was only regulated by NR4A3 at protein level. Furthermore, overexpression of NR4A3 would also enhance EBSS‐induced chondrocytes apoptosis, while knockdown of NR4A3 decreased apoptotic level after EBSS treatment. A pathway study indicated that IL‐1β‐induced NF‐κB activation was enhanced by NR4A3 overexpression and reduced by NR4A3 knockdown. We suggest that NR4A3 plays a pro‐inflammatory role in the development of OA, and we also speculate that NR4A3 mainly regulates cartilage matrix‐degrading gene expression under inflammatory conditions via the NF‐κB pathway.

## 1 INTRODUCTION

Osteoarthritis (OA) is a common degenerative joint disease with loss of cartilage and synovial inflammation.^1^, ^2^ Genetic and environmental factors including gender, obesity and injuries increase the risk for development of OA.^3^ NF‐κB and mitogen‐activated protein kinase (MAPK) are two major pathways involved in OA development.^4^, ^5^, ^6^ Activation of the NF‐κB and MAPK pathways is involved in chondrocyte‐specific gene degradation and cartilage matrix‐degrading enzyme expression in OA development.^7^, ^8^ This progress enhances the catabolic response and exacerbates cartilage matrix degradation.^9^, ^10^ Molecules targeting these pathways may lead to new treatments for OA.

Nuclear receptor subfamily 4 (NR4A) consists of NR4A1 (Nur77), NR4A2 (Nurr1) and NR4A3 (NOR1). These molecules are also known as orphan receptors and are believed to be important regulators involved in cellular function and inflammation reaction.^11^, ^12^ Although NR4A3 is expressed in various cell types, the function of this molecule has poorly been studied. The first study on NR4A3 focused on neurons; NR4A3‐deficient mice showed defects in their inner ear development.^13^ Study of its function in atherosclerosis showed that haematopoietic cell–specific NR4A3 knockout induced atherosclerosis.^14^ Other researchers also identified that NR4A3 is involved in vascular remodelling and arterial disease.^15^, ^16^ Moreover, a recent study reported that overexpression of NR4A3 increased both mRNA and protein levels of vitronectin (VTN) in human vascular smooth muscle cells, and VTN is an adhesive glycoprotein present in the extracellular matrix (ECM).^17^ It is also demonstrated that NR4A receptors modulate MMP9 in vascular muscle cells, and MMP9 is an important extracellular matrix‐degrading enzyme.^18^ Another study reported that NR4A family regulates NF‐κB signalling in myeloid cells.^19^ All these studies indicate that NR4A3 is involved in the regulation of ECM proteins. Furthermore, some other studies recently demonstrated NR4A3 to be involved in promoting apoptosis by inducing expression of pro‐apoptotic genes in cancer cells,^20^, ^21^ while chondrocyte apoptosis is a valid target to modulate cartilage degeneration.^22^

To the best of our knowledge, little is known about the effects and molecular mechanisms of NR4A3 upon OA development. In this study, high expression level of NR4A3 in OA cartilage was observed and its role in OA development was investigated in vitro and in vivo.

## 2 MATERIALS AND METHODS

### 2.1 Reagents

Recombinant rat IL‐1β was purchased from R&D Systems. Dulbecco's modified Eagle's medium (DMEM), penicillin, streptomycin, foetal bovine serum (FBS), Earle's balanced salt solution (EBSS) and 0.25% trypsin were obtained from Gibco BRL. Collagenase II was purchased from Sigma‐Aldrich.

### 2.2 Patients

All patients were selected from the inpatients that underwent total hip arthroplasty (THA) in our facility. Femoral heads were obtained from 10 patients with osteoarthritis and 10 age‐ and sex‐matched patients with femoral neck fractures who underwent THA within 3 days post‐trauma. Femoral heads from patients with femoral neck fractures were used as the normal control. All the patients did not have any other diseases. Cartilage was isolated from those femoral heads for analysis. Each patient signed an informed consent form that was approved by the Ethics Committee of the 2nd Affiliated Hospital, School of Medicine, Zhejiang University, Hangzhou, China.

### 2.3 Chondrocytes harvesting

Sprague Dawley rats (200‐250 g; 6 weeks old) were purchased from the Animal Center of Zhejiang University. Rat chondrocytes were prepared as described in previous studies.^23^, ^24^ All animal studies were conducted in accordance with the Declaration of Helsinki, and the protocol was approved by the Ethics Committee of the 2nd Affiliated Hospital, School of Medicine, Zhejiang University, Hangzhou, China.

### 2.4 Knockdown (KD) experiments using small interfering RNA (siRNA)

NR4A3 siRNA (5′‐GCGUACAGAUAGUCUGAAATT‐3′) and negative control siRNA were obtained from Sangon. P53 siRNA (5′‐GAAGAAAATTTCCGCAAAA‐3′) and its negative control siRNA were purchased from GenePharma. Lipofectamine 2000 transfection reagent (Thermo Fisher Scientific) and Opti‐MEM (Gibco) were used to introduce siRNA into chondrocytes according to the manufacturer's protocol. NR4A3 siRNA‐transfected cells and negative control siRNA‐transfected cells were defined as siNR4A3 and siControl. And P53 siRNA‐transfected cells and its negative control siRNA‐transfected cells were defined as siP53 and siNC.

### 2.5 Overexpression (OE) experiments using lentiviral particles

Lentivirus‐overexpressing NR4A3 particles and overexpressing control particles were purchased from GenePharma. For infections, chondrocytes were incubated with lentiviral particles and polybrene (1 μg/mL) in growth medium. After 12 hours, the infection medium was replaced by growth medium. The expression of NR4A3 and infection efficiency was evaluated by quantitative real‐time PCR, immunofluorescence and Western blot after 72 hours. NR4A3‐overexpressed cells and overexpressing control cells were defined as Len‐OE and Len‐NC.

### 2.6 RNA extraction and real‐time PCR

RNAiso reagent purchased from Takara was used to extract RNA from chondrocytes. Total RNA was reverse transcribed into cDNA (cDNA synthesis kit, Takara). Power SYBR Green PCR Master Mix (Takara) was used for real‐time PCR with an ABI StepOnePlus System (Applied Biosystems). Sequences of the primers used in this study are presented in Table 1. GAPDH was used as an endogenous control. A 10‐μL reaction mixture was used, and the programme included 1 cycle of denaturation at 95°C for 1 minute and 40 cycles of denaturation at 95°C for 15 seconds, primer annealing and extension at 63°C for 25 seconds, followed by melting curve analysis. The data were analysed for fold difference using the formula 2^−ddCT^.

**Table 1:** Primers used for real‐time PCR

Gene	Forward	Reverse
Human NR4A3	CCCATACATGCATGACTCAATCAG	CCAAGGTCCATGGTCAGCTT
Human GAPDH	TTTGCGTCGCCAGGTGAAGA	GTGACCAGGCGCCCAATAC
Rat Nr4A3	AAGACGGAACCTCCACAGAAC	GTCGGGATAGGCGAAGCAG
Rat MMP3	CAGGCATTGGCACAAAGGTG	GTGGGTCACTTTCCCTGCAT
Rat MMP9	GATCCCCAGAGCGTTACTCG	GTTGTGGAAACTCACACGCC
Rat MMP13	GCAAACCCTGCGTATTTCCAT	GATAACCATCCGAGCGACCTTT
Rat Collagen II	GAGTGGAAGAGCGGAGACTACTG	GTCTCCATGTTGCAGAAGACTTTCA
Rat sox9	CCAGCAAGAACAAGCCACAC	CTTGCCCAGAGTCTTGCTGA
Rat COX2	GAGAGATGTATCCTCCCACAGTCA	GACCAGGCACCAGACCAAAG
Rat INOS	CCTTACGAGGCGAAGAAGGACAG	CAGTTTGAGAGAGGAGGCTCCG
Rat GAPDH	GCAAGTTCAACGGCACAG	CGCCAGTAGACTCCACGAC

### 2.7 Western blot analysis

Radioimmunoprecipitation assays (RIPA, Bosterbio) containing protease and phosphatase inhibitors were used to prepare cell extracts. Equal amounts of cell extracts from different samples were separated by 10% SDS‐PAGE and then electrotransferred to polyvinylidene difluoride membranes. After blocking for 2 hours with 5%‐10% bovine serum albumin (BSA, Sigma‐Aldrich), the membranes were blotted with primary antibodies at 4°C for 12‐20 hours and then incubated for 1 hour with secondary antibodies (depending on the origin of the primary antibodies). In this study, antibodies against matrix metalloproteinase (MMP)‐3 (Abcam, ab52915), MMP‐9 (Abcam, ab76003), INOS (R&D Systems, MAB9502), COX‐2 (Cell Signaling Technology, #12282), collagen 2 (Abcam, ab188570), sox9 (Abcam, ab185966), P53 (Abcam, ab26), NR4A3 (Abcam, ab94507), GAPDH (Santa Cruz Biotechnology, sc‐32233), NF‐κB p65 (Cell Signaling Technology, #4764S), phosphor‐NF‐κB p65 (Cell Signaling Technology, #3031), β‐actin (Abcam, ab8226), BAX (Proteintech, 50599), caspase 3 (Abcam, ab13847), cleaved caspase 3 (Abcam, ab2302), caspase 9 and cleaved caspase 9 (Abcam, ab184786), IκBα (Cell Signaling Technology, #4814), phosphor‐SPAK/JNK (Cell Signaling Technology, #9255), SPAK/JNK (Cell Signaling Technology, #9252), phosphor‐Erk1/2 (Cell Signaling Technology, #4370), Erk1/2 (Cell Signaling Technology, #4695), phosphor‐p38 Mapk (Cell Signaling Technology, #4511) and p38 Mapk (Cell Signaling Technology, #8690) were used. GAPDH and β‐actin worked as endogenous controls.

### 2.8 Terminal deoxynucleotidyl transferase‐mediated dUTP nick‐end labelling (TUNEL) staining

Terminal deoxynucleotidyl transferase‐mediated dUTP nick‐end labelling staining was used to detect the apoptotic levels after different treatments. After fixed with 4% PFA for 15 minutes, rat chondrocytes were washed with PBS for three times and permeabilized with 0.3% Triton X‐100 in PBS for 3 minutes. After that, cells were stained by TUNEL staining kit according to the manufacturers' protocol and subsequently counterstained with DAPI for 5 minutes. Images in different groups were observed by a immunofluorescence microscope. Two TUNEL staining kits (C1086 presents green fluorescence and C1089 presents red fluorescence) from Beyotime (Shanghai, China) were used in this study.

### 2.9 Immunofluorescence microscopy

Expression and location of proteins were evaluated by immunofluorescence microscopic analysis. Chondrocytes cultured on glass coverslips were fixed in 4% paraformaldehyde for 10 minutes and permeabilized for 5 minutes with 0.1% v/v Triton X‐100. Then, cells were incubated with primary antibody at 4°C overnight, washed and then incubated with fluorochrome‐conjugated secondary antibodies for 2 hours in the dark. Coverslips were mounted onto glass slides using DAPI‐containing mounting medium.

### 2.10 Luciferase reporter gene analysis

Cells were first stably transfected with a luciferase reporter construct (Nf‐κb‐Luc, Promega). Then, transfected cells were stimulated with IL‐1β (10 ng/mL) for 6 hours. Subsequently, the luciferase assay system (Promega) was used to measure luciferase activity.

### 2.11 Animal experiments

Surgical excision of the medial meniscus (MM) on the knee joints was used to develop a rat model of OA. Firstly, Sprague Dawley rats (200‐250 g; 6 weeks old) were anaesthetized by pentobarbital (40 mg/kg). We used a medial para‐patellar approach to expose the knee. A medial capsular incision was made, and the patella was laterally retracted to expose the medial meniscus. Then, the medial meniscus was removed. After that, operational wound was closed in layers by sutures: The incision of medial capsular was firstly sutured by absorbable sutures, and then, the skin was closed. A sham surgery was performed in Sham group rats using the same surgical approach without remove of medial meniscus.

A total of 75 rats were used in this study, and they were randomly divided into five groups (15 rats in each group). The rats in Sham group received sham surgery as blank control. The other 60 rats received surgical remove of medial meniscus and were regarded as OA rats. Twenty μL lentivirus‐overexpressing NR4A3 particles (1 × 10^8^TU/mL) were injected intra‐articularly in OA rats every 2 weeks in the Lenti‐OE group since 1 week post‐surgery. Lentivirus‐control particles at the same dose were injected intra‐articularly in OA rats in the Lenti‐NC group. Lentivirus‐shNR4A3 particles at the same dose were injected intra‐articularly in OA rats in the Lenti‐KD group. Lentivirus‐shNR4A3 particles were prepared by Cyagen Biosciences Inc The sequence (sense: 5′‐GCGUACAGAUAGUCUGAAATT‐3′, loop: CTCGAG, antisense: 5′‐UUUCAGACUAUCUGUACGCTT‐3′) was designed according to the sequence of siRNA used in knockdown experiments in vitro. Lentivirus‐shcontrol particles at the same dose were injected intra‐articularly in OA rats in the Lenti‐shCon group. Rats were killed after 4 weeks of treatment, and the knees were preserved in 4% paraformaldehyde solution.

Fixed knees were decalcified and embedded in paraffin, and then sectioned at 5 μm thickness. The sections of the interior joint were stained with safranin O‐fast green (SO), and immunofluorescence analysis was subsequently performed. The OARSI assessment system with grading method (0‐6) was used to evaluate the sections.^25^

### 2.12 Statistical analysis

The results are presented as the mean ± standard deviation of three experiments. Statistical differences were determined with SPSS 12.0 software. One‐way ANOVA with subsequent post hoc Tukey's test was used for multiple comparisons. *P* < .05 was considered indicative of statistical significance.

## 3 RESULTS

### 3.1 High expression of NR4A3 in human OA cartilage compared with normal cartilage

**Figure 1:**
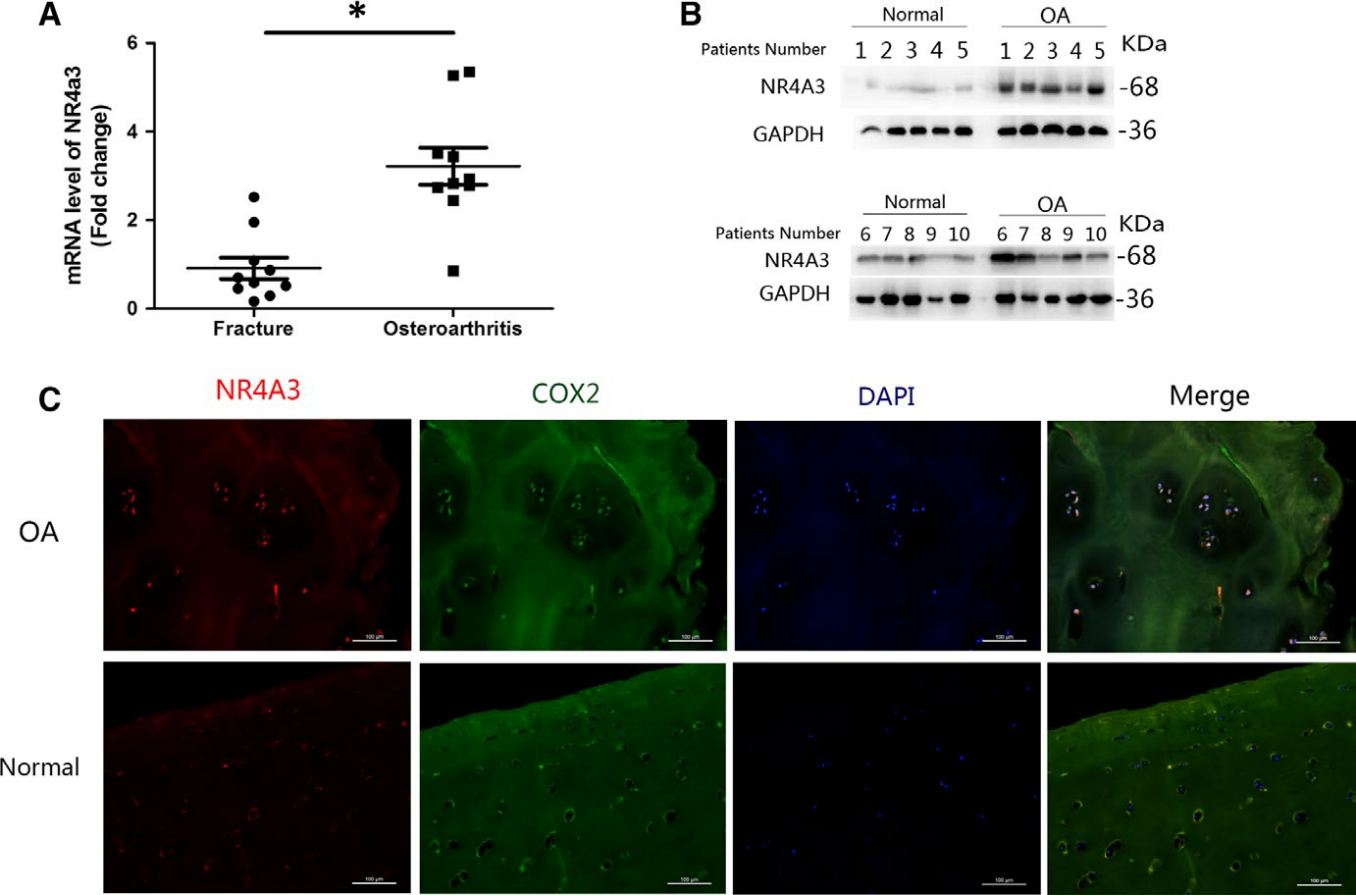
High expression of NR4A3 in human OA cartilage. Cartilage from patients suffered from femoral neck fracture was used as control (sample number = 10 per group). The cartilage of femoral heads (OA and femoral neck fracture) was isolated for (A) RT‐PCR, (B) Western blot and (C) immunofluorescence analysis. High expression of NR4A3 in human OA cartilage was detected at both mRNA and protein levels and visualized in immunofluorescence analysis (similar results were obtained from three repeated experiments). *P < .05

Femoral heads were obtained from 10 OA patients and 10 age‐ and sex‐matched patients with femoral neck fractures who underwent THA within 3 days post‐trauma. Femoral heads from patients with femoral neck fractures were regarded as the normal control. The cartilage of those femoral heads was isolated for RT‐PCR and Western blot. As illustrated in Figure 1, NR4A3 was highly expressed in human OA cartilage at both mRNA and protein levels. Furthermore, immunofluorescence analysis was used to visualize NR4A3 and COX‐2, and COX‐2 was used as an indicator of OA. Those results showed high expression of NR4A3 in human OA cartilage.

### 3.2 Effect of NR4A3 overexpression on IL‐1β‐induced inflammation and EBSS‐induced cell apoptosis in rat chondrocytes

**Figure 2:**
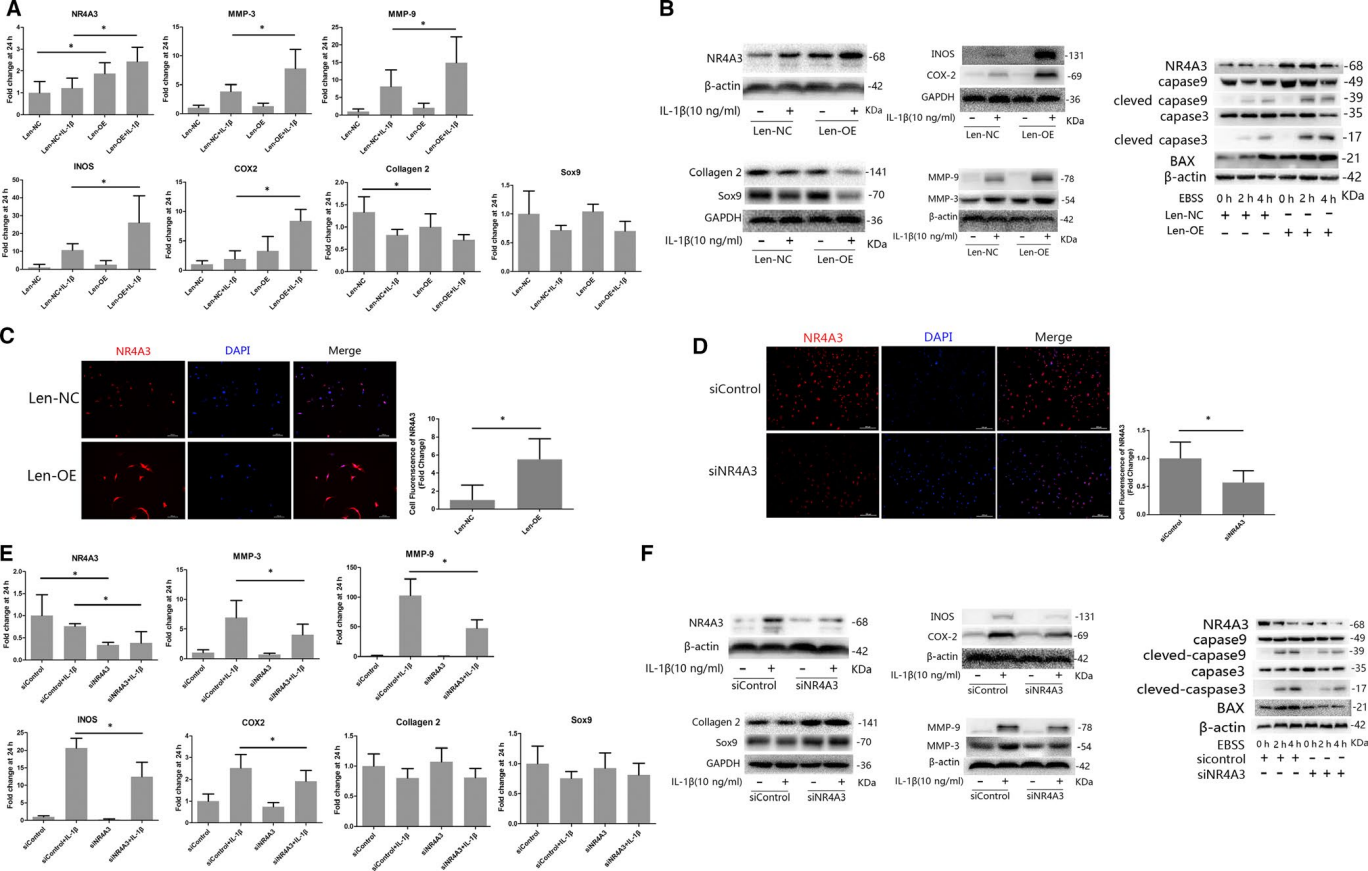
Effect of NR4A3 overexpression and knockdown on IL‐1β‐induced inflammation in rat chondrocytes. The infected chondrocytes (A‐C: overexpression experiments; D‐F: knockdown experiments) were treated with or without IL‐1β (10 ng/ml) for 24 h. (A, E) mRNA level of chondrocyte‐specific genes and matrix‐degrading enzymes was evaluated by real‐time PCR (similar results were obtained from five repeated experiments). (B, F) Protein level of chondrocyte‐specific genes, matrix‐degrading enzymes and pro‐apoptotic genes were evaluated by Western blot (similar results were obtained from 3 repeated experiments). (C, D) NR4A3 was visualized in immunofluorescence analysis (similar results were obtained from three repeated experiments). *P < .05

A lentivirus overexpression system was used in rat chondrocytes to simulate the high expression of NR4A3 observed in human OA cartilage. Firstly, infected chondrocytes (Len‐OE and Len‐NC) were incubated with IL‐1β (10 ng/mL) for 24 hours before analysis, and the effect of NR4A3 overexpression on IL‐1β‐induced chondrocyte‐specific gene degradation and cartilage matrix‐degrading enzyme expression was evaluated by RT‐PCR and Western blot. We also used RT‐PCR, Western blot and immunofluorescence analysis to confirm effective NR4A3 overexpression and infection efficiency in rat chondrocytes (Figure 2A‐C). As shown in Figure 2A,B, the stimulatory effects of IL‐1β on cartilage matrix‐degrading enzyme expression such as MMP‐3, 9, INOS and COX‐2 were enhanced in NR4A3‐overexpressed chondrocytes at both mRNA and protein levels. However, IL‐1β‐induced chondrocyte‐specific gene (collagen 2 and SOX‐9) degradation was only enhanced at protein level; RT‐PCR did not show a significant difference at mRNA level. These results indicated that NR4A3 overexpression increased IL‐1β‐induced matrix‐degrading enzyme expression in rat chondrocytes.

EBSS was used to study the effect of high expression of NR4A3 on chondrocyte apoptosis. Infected chondrocytes (Len‐OE and Len‐NC) were incubated with EBSS for 0 hour, 2 hours or 3 hours before analysis. As shown in Figure 2B, expression of BAX and activation of caspase 3/9 induced by EBSS were enhanced in NR4A3‐overexpressed chondrocytes. Terminal deoxynucleotidyl transferase‐mediated dUTP nick‐end labelling assay (Figure S1A) also showed enhanced apoptosis of NR4A3‐overexpressed chondrocytes.

### 3.3 Effect of NR4A3 knockdown on IL‐1β‐induced inflammation and EBSS‐induced cell apoptosis in rat chondrocytes

To confirm the findings of the overexpression experiment, we used siRNA‐mediated knockdown of NR4A3 to study the effect of NR4A3 knockdown on IL‐1β‐induced inflammation in rat chondrocytes in vitro. Infected chondrocytes (siNR4A3 and siControl) were incubated with IL‐1β (10 ng/mL) for 24 hours before analysis (Figure 2D‐F). The results showed that IL‐1β‐induced cartilage matrix‐degrading enzyme expression was decreased by siRNA‐mediated knockdown of NR4A3 at both mRNA and protein levels. NR4A3 knockdown also decreased IL‐1β‐induced chondrocyte‐specific gene degradation at protein level, but no significant decrease at mRNA level was observed. In cell apoptosis experiments, inhibited apoptosis was observed in siNR4A3‐infected chondrocytes (Figure 2E and Figure S1B). Thus, NR4A3 knockdown decreased IL‐1β‐induced inflammation and EBSS‐induced cell apoptosis in rat chondrocytes in vitro.

### 3.4 Effect of intra‐articular NR4A3 overexpression and knockdown on the rat OA model

**Figure 3:**
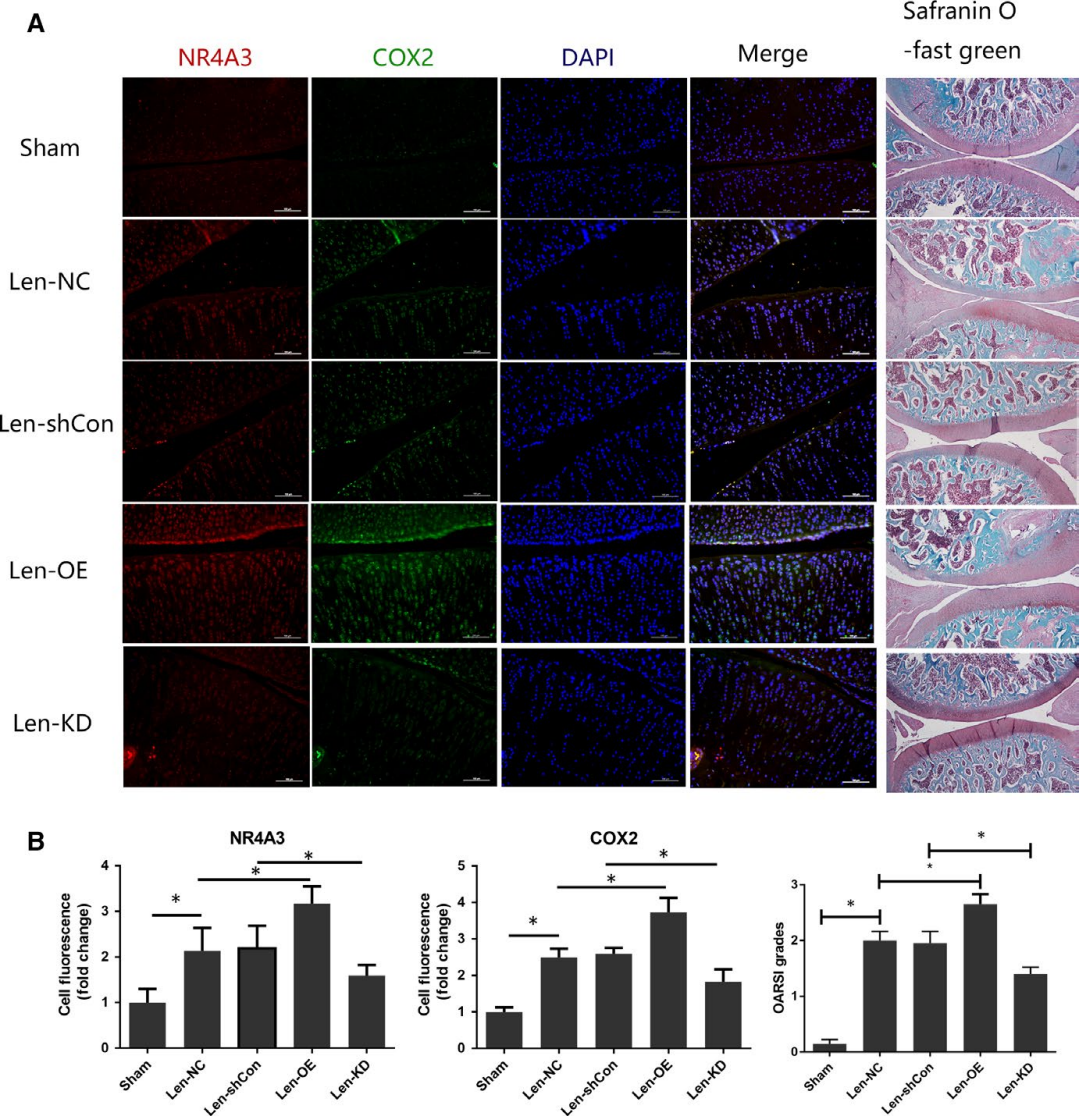
Effect of intra‐articular NR4A3 overexpression and knockdown on rat OA model. Seventy‐five rats were used here, and they were randomly divided into five groups (15 rats in each group). The rats in the Sham group received sham surgery as blank control. The other 60 rats received surgical remove of medial meniscus and were regarded as OA rats. Twenty μL lentivirus‐overexpressing NR4A3 particles (1 × 10^8^TU/mL) were injected intra‐articularly in OA rats every 2 wk in the Lenti‐OE group 1 wk post‐surgery. Lentivirus‐control particles at the same dose were injected intra‐articularly in OA rats in the Lenti‐NC group. Lentivirus‐shNR4A3 particles at the same dose were injected intra‐articularly in OA rats in the Lenti‐KD group. Lentivirus‐shcontrol particles at the same dose were injected intra‐articularly in OA rats in the Lenti‐shCon group. (A) Microscopic images of safranin O‐stained and NR4A3‐ and COX‐2‐stained immunofluorescence analysis of rat knee joint sections. (B) Quantitation of immunofluorescence analysis (obtained from experiments of 5 knees each group) and OARSI grading system (0‐6) (obtained from experiments of 10 knees each group) was used to evaluate the sections. *P < .05

To evaluate the role of NR4A3 in cartilage degeneration in vivo, the rat OA model was induced by surgical excision of MM. Lentivirus‐overexpressing NR4A3 particles (Lenti‐OE) and lentivirus‐shNR4A3 particles (Lenti‐KD) were injected intra‐articularly into OA rats to induce NR4A3 overexpression or knockdown. Respectively, in the knee joints, lentivirus‐control particle (Lenti‐NC)‐injected knees were used as the control for overexpression experiment, and lentivirus‐shcontrol particle (Len‐shCon)‐injected knees were used as control for knockdown experiment. Immunofluorescence analysis of NR4A3 was used to confirm effective infection in the joints. SO staining and immunofluorescence analysis of COX‐2 were used to evaluate the development of OA. Terminal deoxynucleotidyl transferase‐mediated dUTP nick‐end labelling staining was used to evaluate the apoptosis of chondrocytes. The result of the immunofluorescence analysis showed that intra‐articular injection of lentivirus could achieve effective infection in the knee, and more COX‐2 protein was found in the Len‐OE group than Len‐NC and less COX‐2 in the Len‐KD group than Len‐shCon. SO staining revealed that the knees of Len‐KD rats showed healthier cartilage and lower OARSI grade than Len‐shCon rats, while sections of Len‐OE knees showed disrupted and discontinuous cartilage with the highest OARSI grade (Figure 3). Terminal deoxynucleotidyl transferase‐mediated dUTP nick‐end labelling staining showed that apoptotic chondrocytes in Len‐OE group were increased compared to Len‐NC and treatment of Len‐KD decreased apoptosis level (Figure S2). Taken together, these results demonstrated that NR4A3 plays pro‐inflammatory and pro‐apoptotic roles in a rat model of OA.

### 3.5 Effect of NR4A3 overexpression on IL‐1β‐induced NF‐κB and MAPK pathway activation in rat chondrocytes

**Figure 4:**
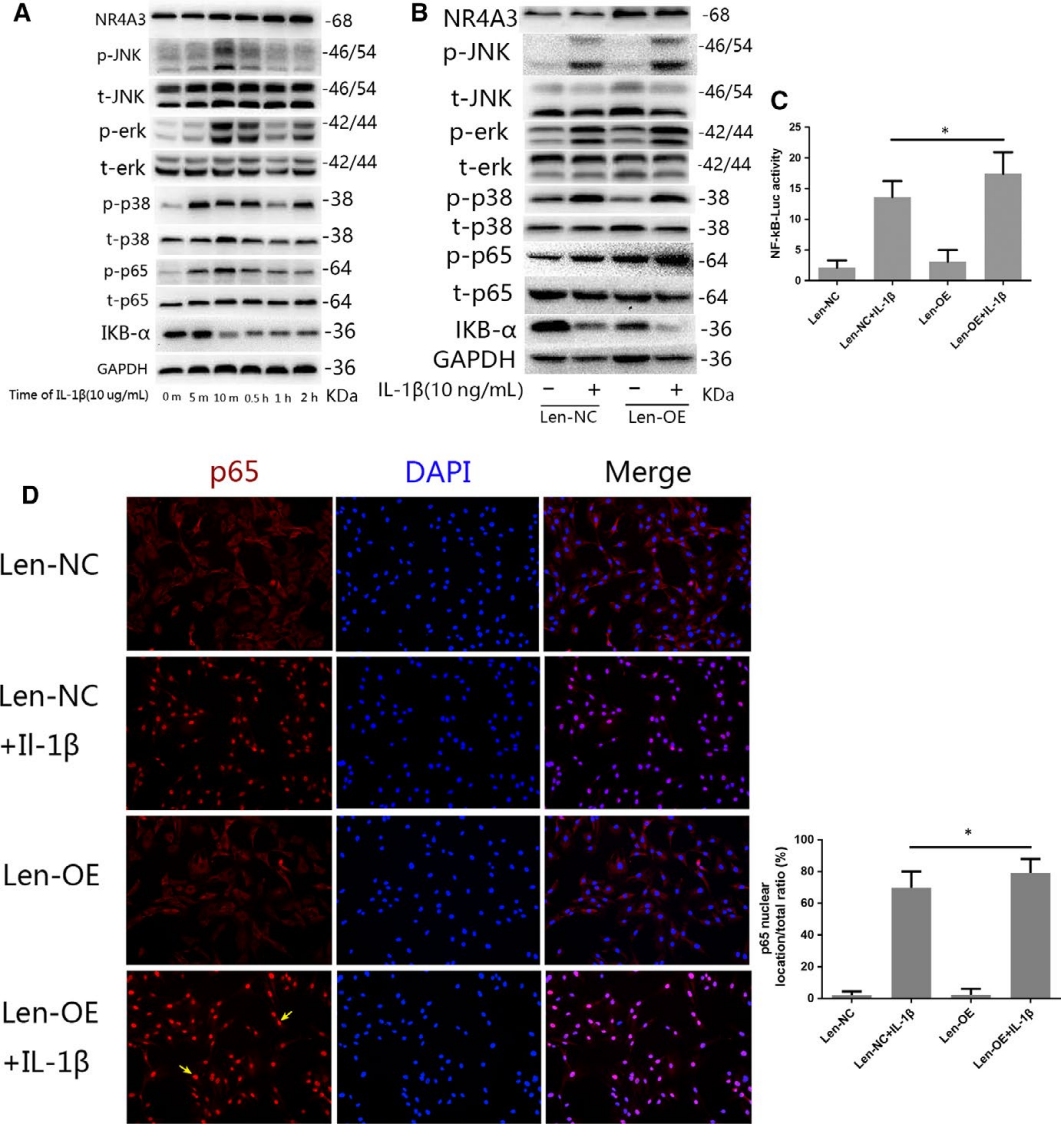
Effect of NR4A3 overexpression on IL‐1β‐induced NF‐κB and MAPK pathway activation in rat chondrocytes. (A) A time‐based experiment was used to affirm a proper duration of IL‐1β stimulation on rat chondrocytes for the analysis of pathway activation (similar results were obtained from 3 repeated experiments). The NF‐κB pathway was evaluated by detecting the phosphorylation of p65, and degradation of IKB‐α, while activation of the MAPK pathway was investigated by detecting the phosphorylation of p38, Erk and JNK through Western blot analysis. Significant activation of the NF‐κB and MAPK pathways was observed at 10 min, and therefore, 10 min was used as the duration of IL‐1β stimulation in the following experiments. (B) NR4A3‐overexpressed or control chondrocytes were incubated with IL‐1β (10 ng/ml) for 10 min, and activation of NF‐κB and MAPK pathways was investigated through Western blot analysis (similar results were obtained from three repeated experiments). (C) Nuclear translocation of p65 was visualized by immunofluorescence analysis (similar results were obtained from three repeated experiments); yellow arrows were used to illustrate enhanced nuclear translocation of p65. (D) Activation of NF‐κB was also detected by luciferase reporter gene analysis (similar results were obtained from 5 repeated experiments). *P < .05

To explore the underlying mechanism of the pro‐inflammatory effect of NR4A3, the NF‐κB and MAPK pathways were investigated in this study. Firstly, we designed a time‐based experiment to affirm the proper duration of IL‐1β stimulation on rat chondrocytes. The NF‐κB pathway was evaluated by detecting the phosphorylation of its key effector, p65 and degradation of IKB‐α, while activation of the MAPK pathway was investigated by detecting the phosphorylation of p38, Erk and JNK through Western blot analysis. Significant activation of the NF‐κB and MAPK pathways was observed at 10 minutes, and therefore, 10 minutes was used as the duration of IL‐1β stimulation in the following experiments (Figure 4A).

Infected chondrocytes were incubated with IL‐1β (10 ng/mL) for 10 minutes before analysis. The results of Western blots demonstrated that overexpression of NR4A3 significantly increased the phosphorylation level of p65 and degradation of IKB‐α, but had no significant effect on MAPK pathways (Figure 4B). Furthermore, the immunofluorescence analysis showed that IL‐1β‐induced nuclear translocation of p65 was enhanced by overexpression of NR4A3 (Figure 4D). The luciferase reporter gene also revealed that activation of the NF‐κB pathway was enhanced by NR4A3 overexpression (Figure 4C). Our data demonstrated that NR4A3 overexpression effectively enhanced the IL‐1β‐induced NF‐κB pathway activation in rat chondrocytes, while having no significant effect upon the MAPK pathway.

**Figure 5:**
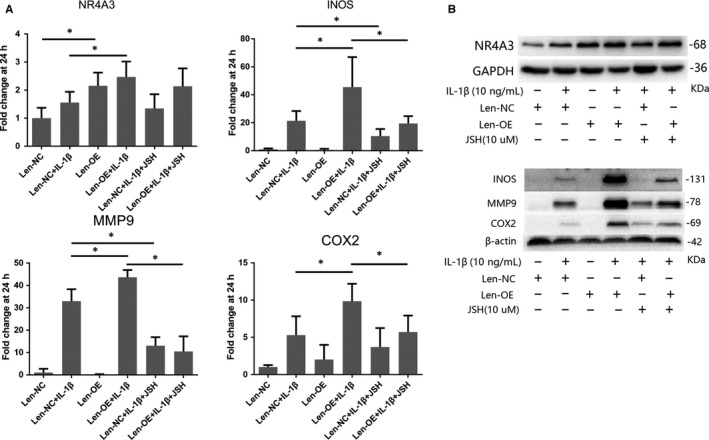
Effect of JSH‐23 on pro‐inflammatory effect of NR4A3 overexpression in rat chondrocytes. NR4A3‐overexpressed or control chondrocytes were treated with or without NF‐κB inhibitor JSH‐23 (10 μmol/L) before IL‐1β stimulation (10 ng/mL). (A) mRNA level of NR4A3, INOS, COX2 and MMP9 was detected by real‐time PCR (similar results were obtained from five repeated experiments). (B) Protein level of NR4A3, INOS, COX2 and MMP9 was evaluated by Western blot analysis (similar results were obtained from 3 repeated experiments). *P < .05

Then, we performed pathway inhibition experiment to confirm the involvement of NF‐κB pathway. The NF‐κB inhibitor JSH‐23 was used in rat chondrocytes transfected with lentivirus‐overexpressing NR4A3 particles under stimulation of IL‐1β. Results of real‐time PCR (Figure 5A) and Western blot (Figure 5B) showed that up‐regulation of matrix‐degrading enzyme expression caused by NR4A3 overexpression upon IL‐1β stimulation was significantly saved by JSH‐23, suggesting that NF‐κB pathway is involved in pro‐inflammatory effect of NR4A3 overexpression.

### 3.6 Effect of NR4A3 knockdown on IL‐1β‐induced NF‐κB and MAPK pathway activation in rat chondrocytes

**Figure 6:**
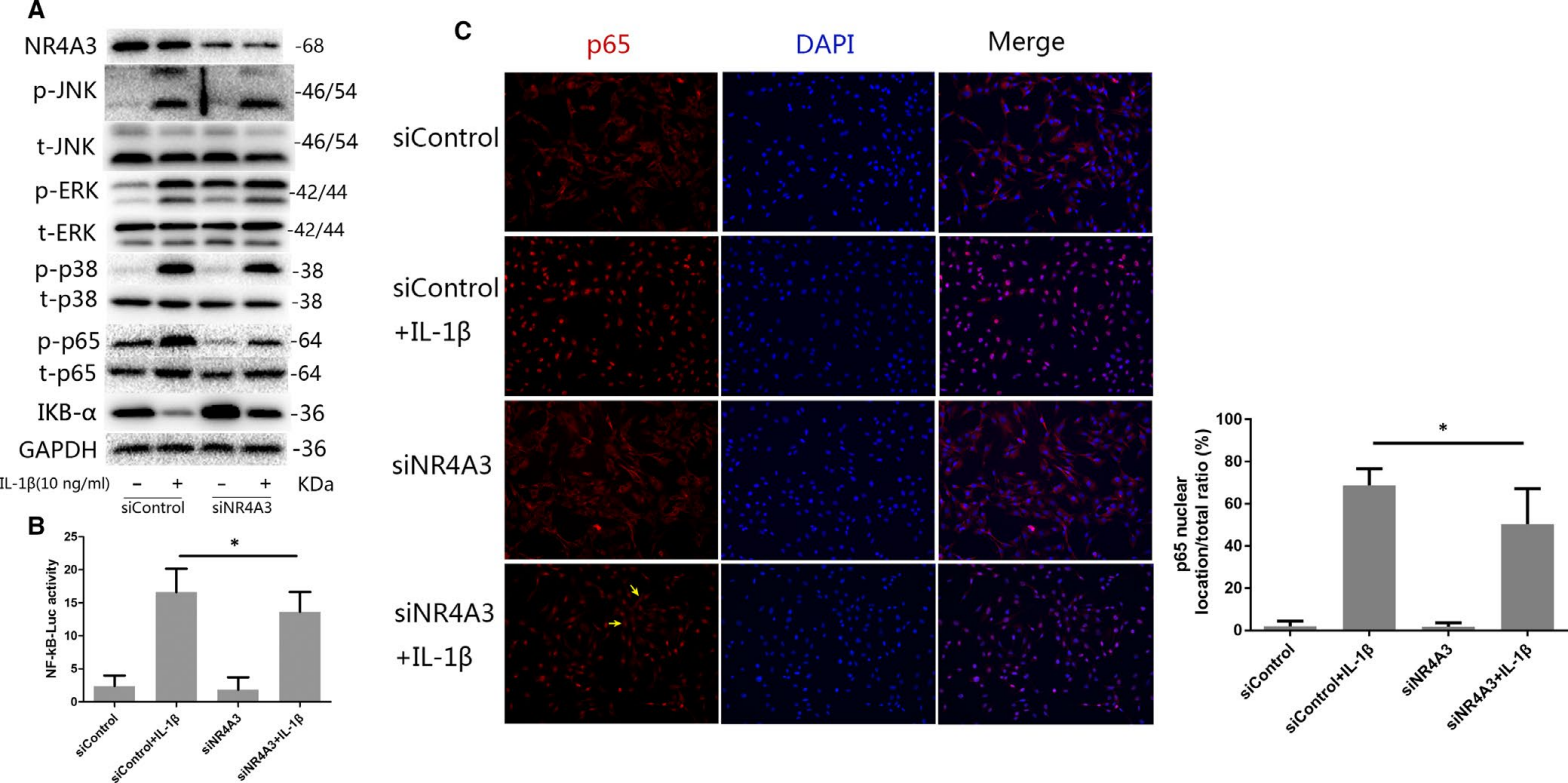
Effect of NR4A3 knockdown on IL‐1β‐induced NF‐κB and MAPK pathway activation in rat chondrocytes. (A) NR4A3‐knockdown or control chondrocytes were incubated with IL‐1β (10 ng/mL) for 10 min, and the NF‐κB pathway was evaluated by detecting the phosphorylation of its key effector p65 and degradation of IKB‐α, while activation of the MAPK pathway was investigated by detecting the phosphorylation of p38, Erk and JNK through Western blot analysis (similar results were obtained from 3 repeated experiments). (B) Nuclear translocation of p65 was visualized by immunofluorescence analysis (similar results were obtained from three repeated experiments); yellow arrows were used to illustrate inhibited nuclear translocation of p65. (C) Activation of NF‐κB was also detected by luciferase reporter gene analysis (similar results were obtained from five repeated experiments). *P < .05

We also used siRNA‐mediated knockdown of NR4A3 to confirm the findings of the overexpression signalling study. As shown in Figure 6, the results demonstrated that NR4A3 knockdown significantly decreased the phosphorylation level of p65 and degradation of IKB‐α, but had no significant effect on MAPK pathways. The immunofluorescence analysis showed that nuclear translocation of p65 was inhibited by NR4A3 knockdown, and luciferase reporter gene also confirmed the findings. These results indicate that NR4A3 knockdown inhibited IL‐1β‐induced NF‐κB pathway activation in rat chondrocytes, while having no significant effect on MAPK pathways.

## 4 DISCUSSION

Our current understanding of OA suggests that it is a disease of the joints with cartilage degeneration, synovial inflammation and subchondral sclerosis.^26^ Degeneration of cartilage plays a central role in OA development, which is caused by an imbalance between cartilage matrix synthesis and degradation. This imbalance is presented as an up‐regulation of matrix‐degrading genes like MMPs and down‐regulation of chondrocyte‐specific genes such as collagen II and Sox‐9.^27^ At the beginning, we showed high expression of NR4A3 in human OA cartilage at both protein and mRNA levels. After that, we used a lentivirus overexpression system to simulate this high expression to study its role in OA. Additionally, considering that there is possible cooperation with other molecules, and overexpression experiments are not simple gain‐of‐function studies,^28^ siRNA‐mediated knockdown of NR4A3 was used to confirm the findings of the overexpression experiments.

To the best of our knowledge, we are the first to identify the pro‐inflammatory role of NR4A3 in OA development. Our data showed that high expression of NR4A3 enhanced matrix‐degrading genes expression at both protein and mRNA levels. Although chondrocyte‐specific gene degradation was also enhanced at protein level, interestingly, overexpression did not alter mRNA level of chondrocyte‐specific genes. High expression of NR4A3 would also enhance EBSS‐induced chondrocytes apoptosis. Knockdown experiments confirmed these findings. Therefore, we suggested that NR4A3 mainly regulates matrix‐degrading gene expression under the inflammatory condition.

Although NR4A3 is expressed in various cell types, its function is not well studied. One previous study reported that NR4A3 modulates the inflammatory response of vascular smooth muscle cells by preventing NF‐κB activation. The data showed that overexpression of NR4A3 would reduce LPS‐induced up‐regulation of cytokines, and siRNA‐mediated NR4A3 knockdown increased the expression of pro‐inflammatory mediators in vascular smooth muscle cells,^29^ which is quite different from the results presented in our study. Furthermore, another study of vascular smooth muscle cells also indicated a different result.^18^ However, other NR4A3 studies have obtained different results as well, such as Qing H et al, who reported that haematopoietic cell‐specific NR4A3 knockout (KO) accelerates atherosclerosis with monocytosis,^14^ while full NR4A3 KO mice showed decreased atherosclerosis according to another study,^30^ suggesting that NR4A3 possesses cell‐specific function.

Chondrocyte apoptosis is also a valid target to modulate cartilage degeneration. Maintenance of extracellular matrix of articular cartilage depends on the chondrocytes. Dysfunction and death of chondrocytes would lead to the failure of articular cartilage.^22^ Previous studies showed that NR4A3 is involved in promoting apoptosis in lymphoma and cancer cells by acting as downstream of P53.^20^, ^21^ Here, our data demonstrated this similar function of NR4A3 in rat chondrocytes (Figure 2 and Figure S3).

MAPKs and NF‐κB are the two major pathways upon which we focused in this study. MAPKs mainly include the extracellular signal‐regulated kinases 1 and 2 (Erk1/2), c‐Jun amino‐terminal kinases (JNK) and p38, and they can be activated by phosphorylation.^31^ In the resting state, p65 is stable with its inhibitor IKB in the cytoplasm. When activated by inflammatory factors, p65 is freed with phosphorylation because of IKB degradation and translocated into the nucleus, where p65 up‐regulates multiple inflammation‐related genes, such as MMPs, COX‐2 and PGE2.^32^, ^33^ Our results revealed that NR4A3 is involved in the regulation of NF‐κB activation in OA. Overexpression of NR4A3 would enhance activation of NF‐κB pathway, and this finding is confirmed by the knockdown experiment. A recent study of NR4A3's role in dendritic cell activation obtained a similar result, where it was revealed that knockdown of NR4A3 in dendritic cells would decrease the synthesis of IKKβ, which is quite important in the degradation of IKB.^28^ However, it is still unknown whether NF‐κB is the direct target of NR4A3; we plan to further investigate the role of NR4A3 in the NF‐κB pathway.

In conclusion, our in vitro and in vivo experiments strongly suggest that NR4A3 plays a pro‐inflammatory role in the development of OA, and we also speculate that NR4A3 mainly regulates cartilage matrix‐degrading gene expression under inflammatory conditions via the NF‐κB pathway. Thus, our results suggest that NR4A3 is a potential therapeutic target for the treatment of OA.

## CONFLICT OF INTEREST

No conflicts of interest have been reported by the authors or by any individuals in control of the content of this article.

## AUTHOR CONTRIBUTIONS

All authors listed have made substantial contributions to the study. Lidong Wu, HW, SY, KS, LS, CM and JR took part in the designing of the experiments, contributed reagents, materials and analysis tools. CM, Lingyun Wu, KS, LS and JR run the experiments. CM and Lingyun Wu wrote the manuscript. Lidong Wu and JR also participated in the analysing of the data. All authors read and approved the final manuscript. All the data presented in the figures were obtained from experiments run by CM, Lingyun Wu, KS, LS and JR. In Figures 1 and 2, CM and Lingyun Wu prepared all the panels of RT‐PCR data. LS generated all the Western blot data in Figures 1,2,4, 5, 6. All the data in Figures 1 and 3 from animal experiments and human samples are prepared by CM, Lingyun Wu and LS. KS and CM generated all the immunofluorescence pictures in Figures 2, 4 and 6 and prepared the merged pictures. The data and panels of luciferase assay in Figures 4 and 6 were obtained and prepared by Lingyun Wu and CM. Lidong Wu, Haobo Wu, Shigui Yan and CM checked all the data presented in figures and CM assembled all the figures.
