# Supplementary figures and images for: The pro‐inflammatory effect of NR4A3 in osteoarthritis
Source: J Cell Mol Med. 2019 Nov 7;24(1):930–40. doi: 10.1111/jcmm.14804 (PMC6933326; doi:10.1111/jcmm.14804)

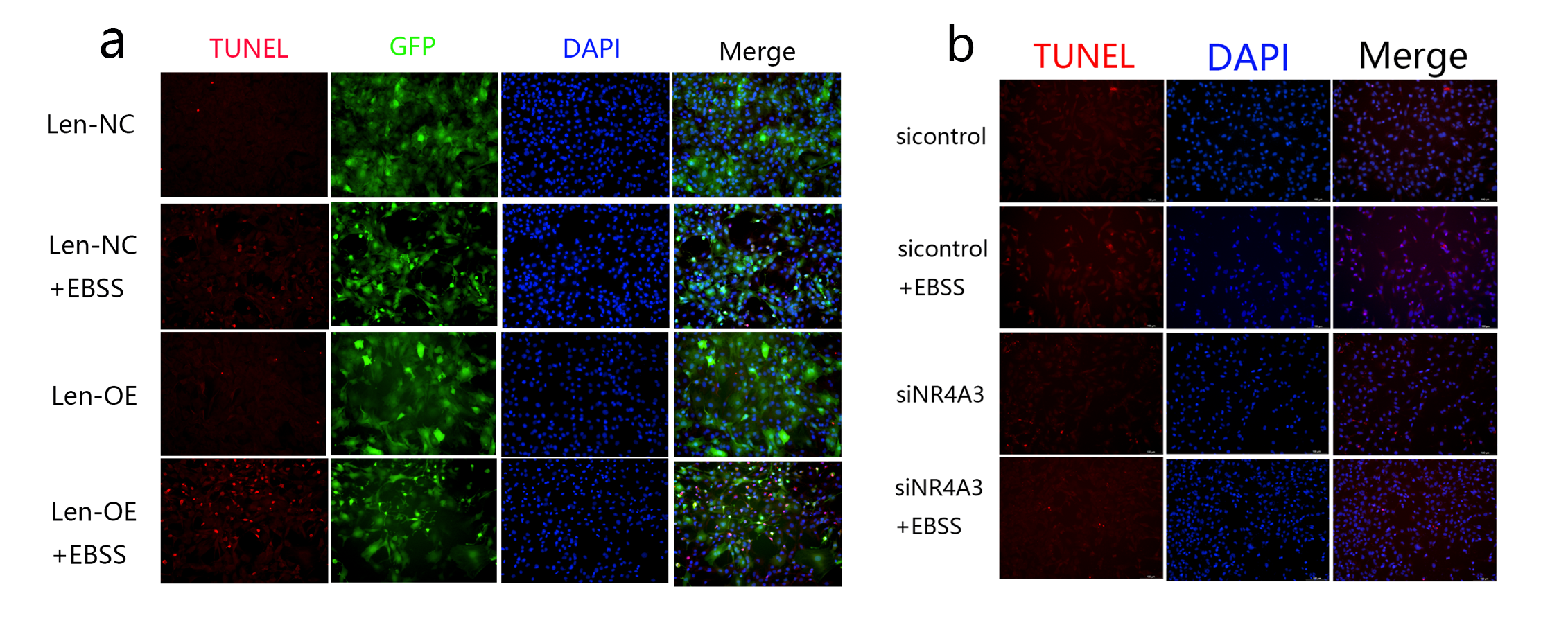

Supplement: Supplementary file 1 [file JCMM-24-930-s001.tif]

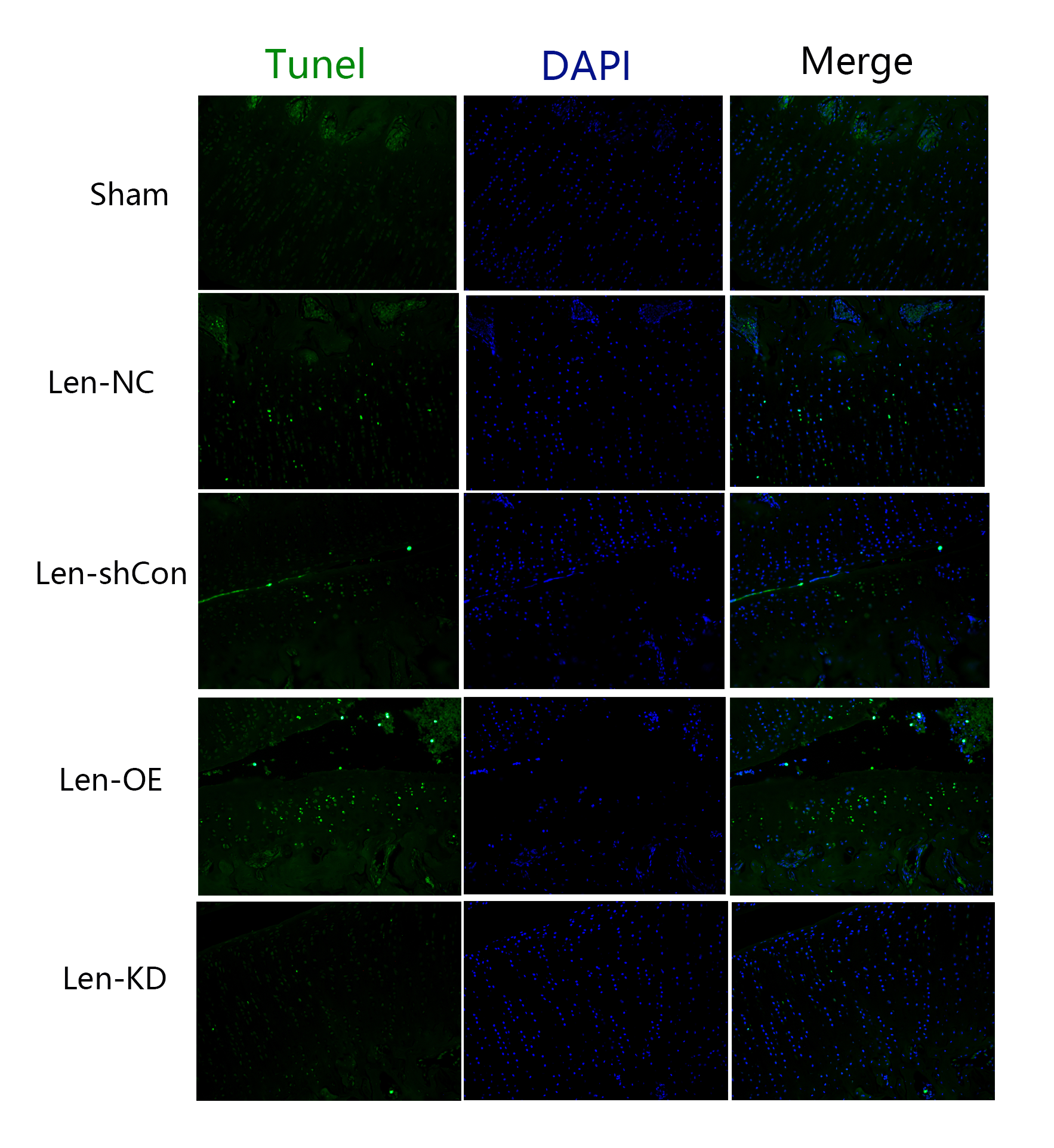

Supplement: Supplementary file 2 [file JCMM-24-930-s002.tif]

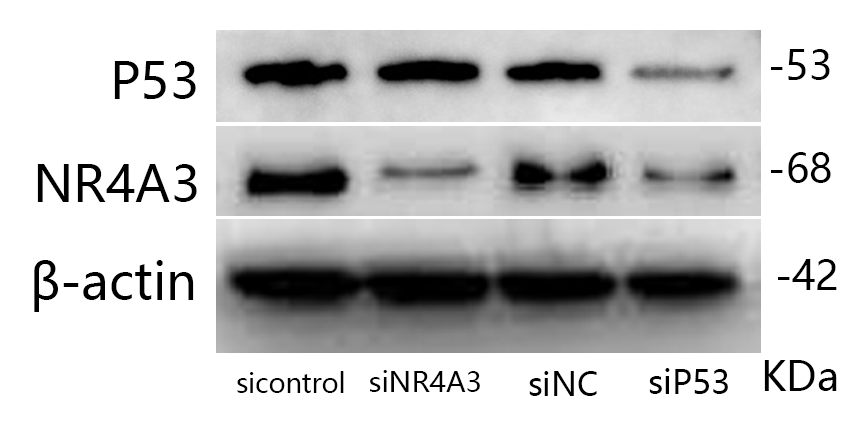

Supplement: Supplementary file 3 [file JCMM-24-930-s003.tif]
